# preon: Fast and accurate entity normalization for drug names and cancer types in precision oncology

**DOI:** 10.1093/bioinformatics/btae085

**Published:** 2024-02-21

**Authors:** Arik Ermshaus, Michael Piechotta, Gina Rüter, Ulrich Keilholz, Ulf Leser, Manuela Benary

**Affiliations:** Institute for Computer Science, Humboldt-Universität zu Berlin, Berlin 10099, Germany; Institute for Computer Science, Humboldt-Universität zu Berlin, Berlin 10099, Germany; Charite Comprehensive Cancer Center, Charite—Universitätsmedizin Berlin, Berlin 10115, Germany; Charite Comprehensive Cancer Center, Charite—Universitätsmedizin Berlin, Berlin 10115, Germany; Institute for Computer Science, Humboldt-Universität zu Berlin, Berlin 10099, Germany; Charite Comprehensive Cancer Center, Charite—Universitätsmedizin Berlin, Berlin 10115, Germany; Core Unit Bioinformatics (CUBI), Berlin Institute of Health, Charite—Universitätsmedizin Berlin, Berlin 10115, Germany

## Abstract

**Motivation:**

In precision oncology (PO), clinicians aim to find the best treatment for any patient based on their molecular characterization. A major bottleneck is the manual annotation and evaluation of individual variants, for which usually a range of knowledge bases are screened. To incorporate and integrate the vast information of different databases, fast and accurate methods for harmonizing databases with different types of information are necessary. An essential step for harmonization in PO includes the normalization of tumor entities as well as therapy options for patients.

**Summary:**

preon is a fast and accurate library for the normalization of drug names and cancer types in large-scale data integration.

**Availability and implementation:**

preon is implemented in Python and freely available via the PyPI repository. Source code and the data underlying this article are available in GitHub at https://github.com/ermshaua/preon/.

## 1 Introduction

Precision oncology (PO) considers the molecular makeup of cancer patients for therapy decisions and promises better-targeted therapies. It requires extensive knowledge bases about associations of molecular features, cancer types, and drugs, which are typically created by integrating multiple specialized databases to leverage international community efforts (Starlinger *et al.* 2018, Pallarz *et al.* 2019, Wagner *et al.* 2020). Such an integration requires the accurate normalization of biomedical entities from different databases into a common ontology (Sharp 2017).

Based on French and McInnes (2023), two major types of entity normalization have been established, namely multi-pass (or multi-step) algorithms and deep learning algorithms. Multi-pass algorithms include abbreviations, synonyms, and/or derivational variants that reflect the use of words in written text (French and McInnes 2023). For example, for the UMLS metathesaurus, different multi-pass approaches have been established to extract concepts from text, ranging from MetaMap (Aronson 2001) to QuickUMLS (Soldaini and Goharian 2016). A similar multi-pass approach has been introduced for the domain specific normalization task of disorder mentions in clinical reports and biomedical abstracts (D’Souza and Ng 2015), but is not available anymore. Other entity normalization tools, such as BERN (Kim *et al.* 2019) or BioSyn (Sung *et al.* 2020) use neural networks for normalization. These tools take contextual information into account and apply semantic matching. This approach leads to a high performance concerning accuracy but is generally very compute-intensive.

For the integration of databases, the normalization relies exclusively on name features because the entities originate from database columns rather than natural language sentences. In addition, as databases may contain a substantial number of entities, a normalization algorithm must be both fast and accurate.

**Figure 1.**
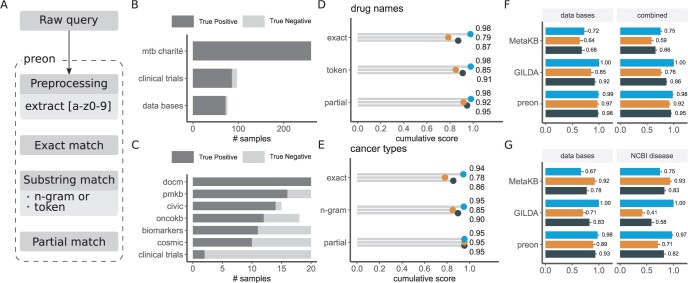
(A) The term normalization workflow established in preon. The normalization consists of a multi-step matching process. Overview of the gold standard for (B) drug names and (C) cancer types. The increase in precision (blue), recall (orange), and F1 score (dark gray) when using exact, token, and partial matching is depicted in (D) for drug name normalization (combined data set) and in (E) for cancer types (data bases). (F, G) performance comparison using preon and the normalization presented in MetaKB (Wagner *et al.* 2020) and GILDA (Gyori *et al.* 2022) for drug names and cancer types, respectively. Mean precision (blue), recall (orange), and F1-score (dark gray) are shown for different gold standards (bootstrapping with *n* = 100) and error bars depict standard deviation.

We present preon, a Python library for drug name and cancer-type normalization in data integration projects. To balance speed and accuracy, preon is based on an efficient multi-step process performing a cascade of matching algorithms of increasing complexity (see Fig. 1A). As preprocessing, preon transforms a given entity name by extracting its alphanumeric characters and applying a lowercase transformation. As a first matching step, preon tries to match the reduced sequence exactly to its preprocessed reference dictionary. If not successful, preon next performs token or n-gram based name matching to allow for different subword orders and slight variations. If still unsuccessful, preon calculates the normalized pairwise edit distances to all reference names. Steps two and three apply individual thresholds to define matches.

With preon, we are focusing on specific reference sets suitable for PO. For example, Disease ontology (DO) allows the distinction between organ-specific and tissue-specific terms for oncology which might have relevant implications for treatment recommendations. Currently, DO is not covered in UMLS and DO concepts related to cancer account for a high number of unmapped concepts with respect to SNOMED CT (Raje and Bodenreider 2017). Based on a similar reasoning, preon uses ChEMBL for drug names which contains bioactivity data covering all stages of the drug discovery process (Mendez *et al.* 2019, Zdrazil *et al.* 2024). Although these reference data sets are specific for the field of PO, preon is flexible and we also provide data loading for DrugBank and MeSH which are part of UMLS.

## 2 Materials and methods

### 2.1 Selecting thresholds for fuzzy matching

In the initial phase, we determined the optimal number of elements for partial matching (for drug names) and the number of tokens for n-gram matching (cancer entities). The use of moderate partial matching thresholds (20%–30%) along with bigrams enhances recall while upholding high precision, as illustrated in Supplementary Fig. S2.

As the partial matching thresholds increase, there is a subsequent rise in the number of identifiers for users to inspect. Therefore, our optimization process not only considered achieving the best precision and recall but also factored in the resulting number of outcomes (see Supplementary Fig. S2). The thresholds are established for two different datasets on cancer entities separately with similar results.

### 2.2 Creating gold standards for benchmarking

There exists a range of biomedical corpora for natural language processing (see French and McInnes 2023 for an overview). These corpora are a great resource for text mining questions but mostly lack the distinct issues in manually curated databases (e.g. spelling issues). As there is no gold standard for drug names and the possibilities of synonyms are extensive, we generated our gold standard using commonly used names from three different types of sources (see Supplementary Table S1). First, we used drug names presented at the molecular tumor board at the Charité Comprehensive Cancer Center. Second, we sampled drug names from the databases Biomarkers, CIViC, oncoKB, and TARGET. And last, we used the semi-structured entries from https://clinicaltrials.gov/ in the column “Intervention” to sample another cohort. From the original data, we removed samples that describe a drug class (e.g. BRAF inhibitor), and common names were matched to the corresponding ChEMBL ID to generate the gold standard (see https://github.com/ermshaua/preon/ and Supplementary Table S1). For the normalization of cancer entities, we relied on two different types of data sources. First, we sampled 20 entities from different databases, respectively, including clinical trials (see https://github.com/ermshaua/preon/ and Supplementary Table S2) and matched them manually with the corresponding entry in DO (Schriml *et al.* 2019). Second, we used NCBI Disease, a data set with abstracts (Doğan *et al.* 2014) in which diseases are annotated with MESH/OMIM-IDs. We used mondo (Shefchek *et al.* 2020) to relate the MESH identifiers with the corresponding ones from DO. Because we are focusing on the normalization of tumor entities, we reduced the dataset by including only diseases from the cancer-related subtree (DOID: 162).

### 2.3 Evaluation

We assessed the impact of the three matching steps in preon by measuring precision, recall, and F1 score. We also evaluated runtimes and compared results to the term normalization performed in MetaKB (Wagner *et al.* 2020), a large integration project for PO. MetaKB performs normalization using mainly web queries with BioThings (Xin *et al.* 2018) and ChEMBL (Mendez *et al.* 2019). Additionally, we compare preon with GILDA (Gyori *et al.* 2022), a tool which employs a scored string matching algorithm, including disambiguation models based on surrounding context. As such, GILDA is more geared toward extraction tasks from texts and has been introduced with a focus on biological terms, as for example genes and gene products or cell lines and tissues.

For drug name normalization, exact matching in preon yields an F1-score of 87%. N-gram matching improves the F1-score by 4pp (percentage points) and edit-distance-based matching adds another 4pp (see Fig. 1D). The best pre-defined similarity threshold for partial matching was established at 20% for our gold standard. In cancer type normalization, exact matching reaches an F1-score of 86%, improved by 4pp through n-gram matching and further 5pp through edit-distance-based matching (Fig. 1E) also using a threshold of 20%.

preon outperforms MetaKB on all measures for drug name normalization (see Fig. 1F), with an increase in F1 of almost 30pp. For cancer type normalization, preon has higher precision yet lower recall than MetaKB for both gold standards (see Fig. 1G). In terms of F1, preon outperforms MetaKB by 15pp in the database data set and is on-par (−1pp) on the NCBI disease data set. GILDA outperforms both MetaKB and preon concerning precision (100%) for drug name normalization as well as cancer entities, but falls back on recall. In terms of F1 score, preon outperforms by at least 6pp.

preon also is very fast because the costly steps two and especially three are only applied when the previous steps found no match, which very often is not the case. preon requires 53 ms on average for normalizing a single drug name and 7 ms for cancer types in the evaluation (see Supplementary Table S5). When applied to full databases, preon requires, for instance, 14s/7s to normalize all 2.8k drug names/3.5k cancer types from CIVIC. In contrast, such a normalization with MetaKB is not feasible as it is implemented as a web service. GILDA demonstrates faster performance than preon, with average query times of less than 1ms for both drug name and cancer type normalization. However, this speed comes at the expense of reduced accuracy.

## 3 Conclusion

preon is an accurate library for normalizing drug names and cancer types and it is fast enough to be applied in large PO integration projects. Critical decisions for building and assessing such a library are the choice of reference library and the construction of the gold standard for evaluation. Regarding reference libraries, DrugBank for drug names and MeSH for cancer types are viable candidates. We provide access to both databases in our implementation and examples of easily exchanging the reference set in preon. Regarding evaluation, our four gold standard datasets have a limitation: the ratios of true negatives to true positives are somewhat arbitrary. However, these ratios significantly impact accuracy and runtime measurements, especially because trying to normalize entity names without a match is costly. We thus believe that an international effort to create appropriate evaluation data would be an important step for the future. Furthermore, we believe that preon’s runtime can be improved further by using advanced indexing techniques for the second and third step (Wandelt *et al.* 2014).

## Supplementary Material

btae085_Supplementary_Data
